# Supplementary material for: Single cell RNA-sequencing of feline peripheral immune cells with V(D)J repertoire and cross species analysis of T lymphocytes
Source: Front Immunol. 2024 Nov 15;15:1438004. doi: 10.3389/fimmu.2024.1438004 (PMC11604454; doi:10.3389/fimmu.2024.1438004)

Figure S1

A

Sample/Age	Mean count RNA per cell	Median count RNA per cell	Mean count features per cell	Median count features per cell
6-month	4338.813	3085.504	1451.185	1355.0
1-year	3743.530	3225.758	1293.321	1225.5
4-years	2832.122	2327.403	1545.825	1417.5
9-years	3706.864	3119.782	1372.430	1525.0

B

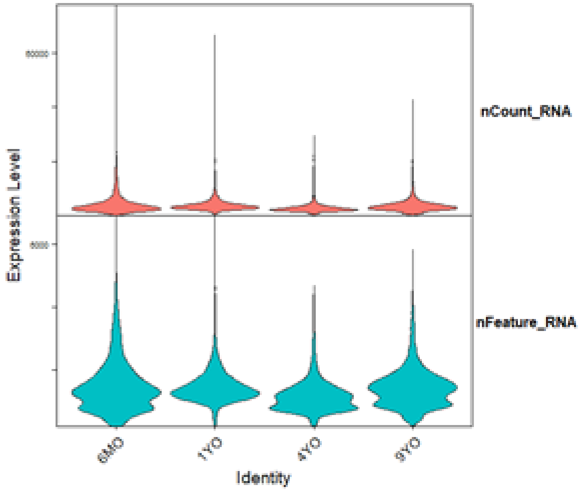

Figure S2

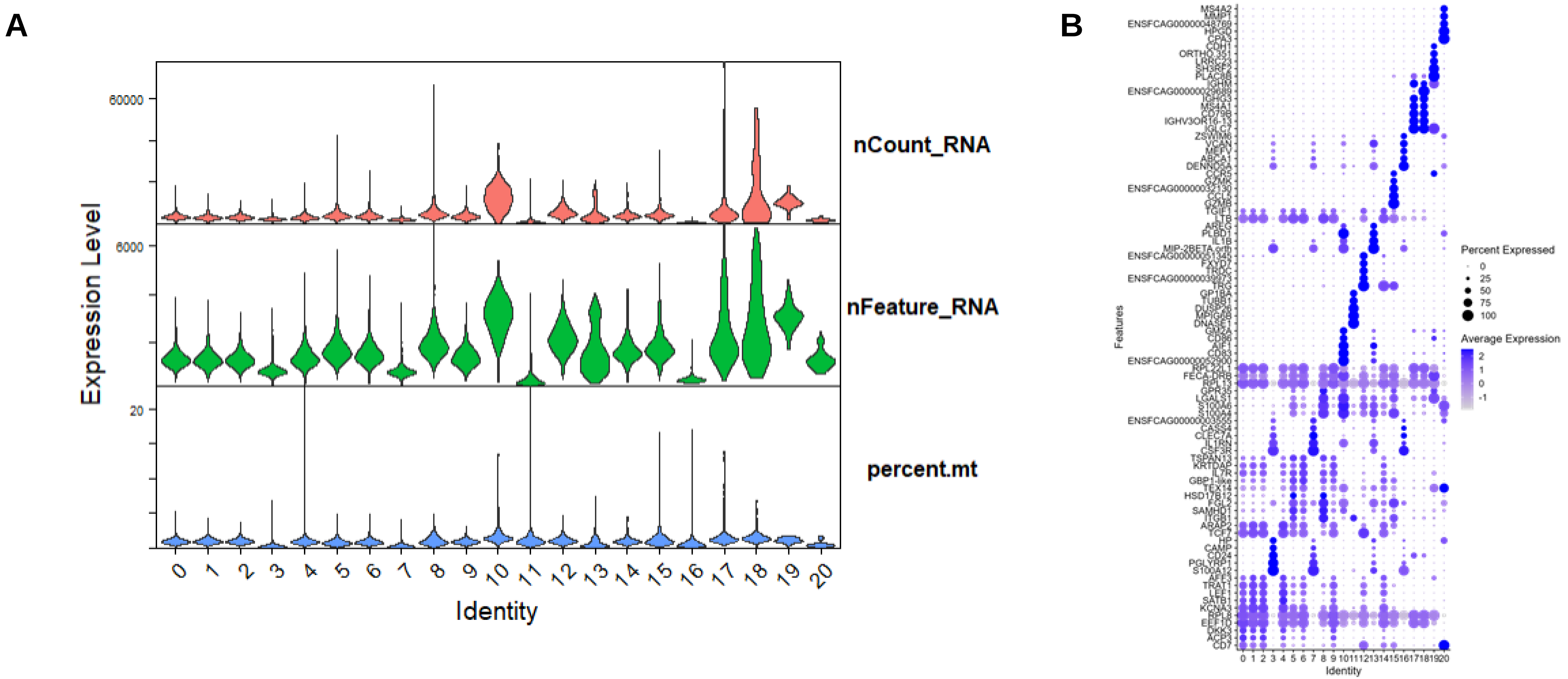
A
B
nCount_RNA
nFeature_RNA
percent.mt
Expression Level
Identity
Features
Percent Expressed
Average Expression

Figure S3

**Module**	**Genes**
Treg	*IKZF2, FOXP3, IL2RA, CTLA4*
Th2	*LGALS3, GATA3, CCR4*
Th1	*IL18R1, IL12RB1, TNF, IFNG, TBX21*
Th17	*RORC, CCR6, KLRB1, IRF4*

Figure S4

A

	**C0**	**C1**	**C2**	**C3**	**C4**
**6MO**	121	82	53	22	12
**4YO**	106	36	13	72	25
**9YO**	399	182	61	28	25

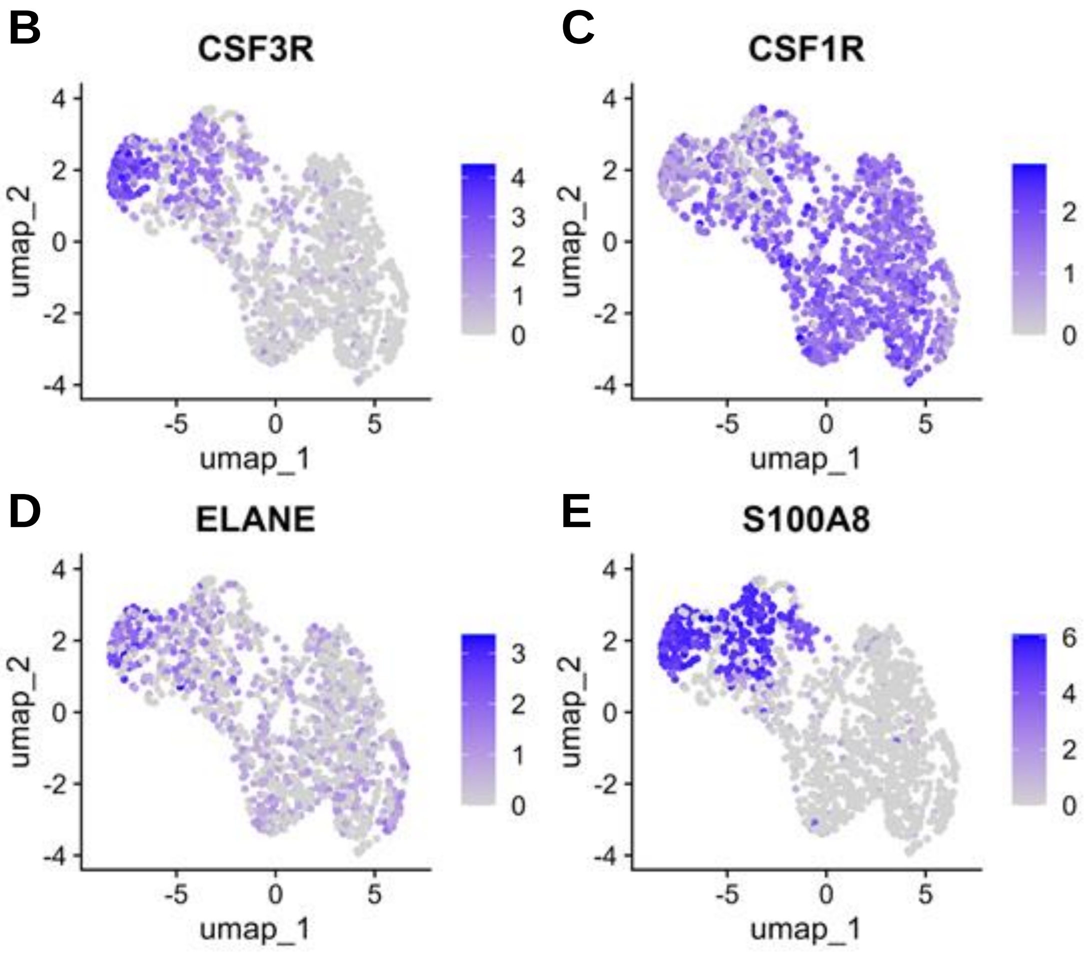

Supplement: Supplementary file 12 [file Presentation1.pdf]
