# Supplementary figures and images for: Single cell RNA-sequencing of feline peripheral immune cells with V(D)J repertoire and cross species analysis of T lymphocytes
Source: Front Immunol. 2024 Nov 15;15:1438004. doi: 10.3389/fimmu.2024.1438004 (PMC11604454; doi:10.3389/fimmu.2024.1438004)

# Gating Strategy

## 22-007 presort

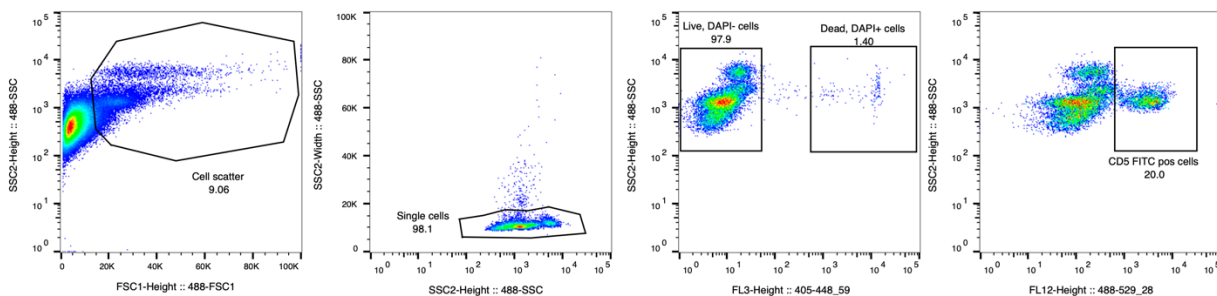

## 21-005 presort

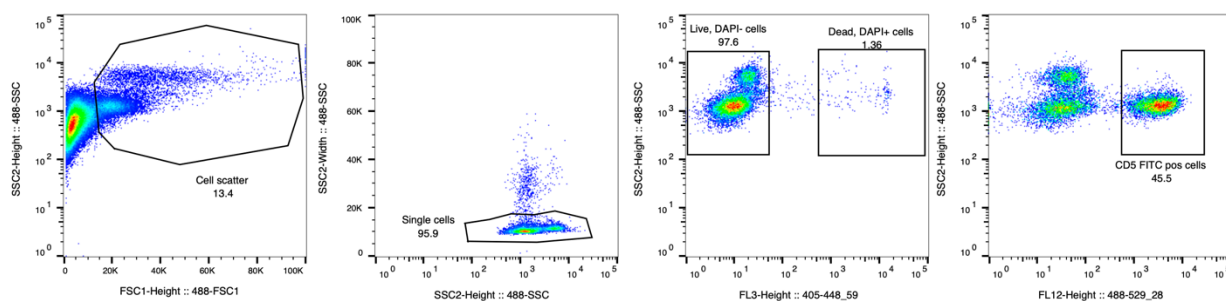

## 18-003 presort

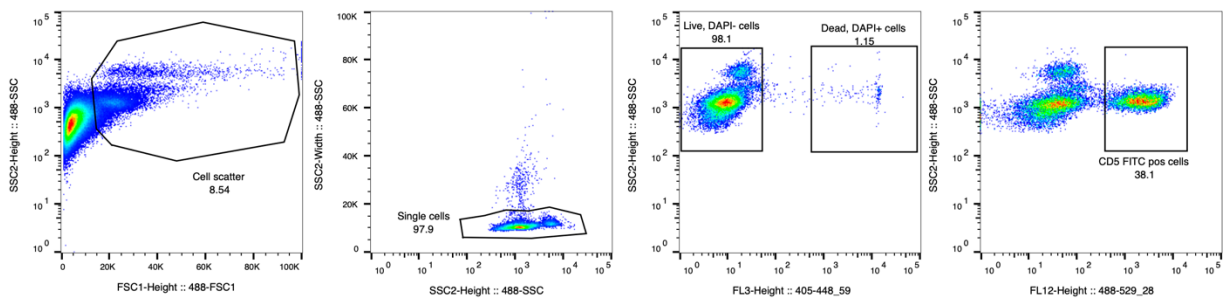

## 13-101 presort

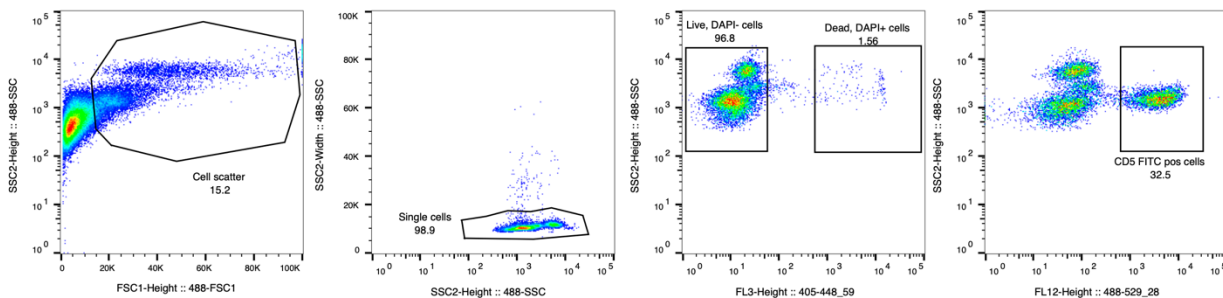

Supplement: Supplementary file 13 [file Presentation2.pdf]
